# 

**Published:** 1908-06-13

**Authors:** 


CONTENTS.

| | PAGE |
|---|---|
| **A New View of Diabetes** | 273 |
| **Deaths after Operations** | 274 |
| **Annotations—** | |
| The Model Prescription—"Preserved" Cream—Enteric Fever in Belfast | 275 |
| **Medical Opinion and Movement** | 276 |
| **Hospital Clinics—** | |
| Tetany. By Edmund Cautley, M.D., F.R.C.P. | 279 |
| **Special Article—** | |
| The Effects of Syphilis upon Phthisis | 282 |
| **Medicine—** | |
| The Sequelæ of Enteric Fever—II. | 283 |
| **Tropical Diseases—** | |
| Filariasis—III. | 284 |
| **Points in Surgery—** | |
| Some Aspects of Intestinal Obstruction | 285 |
| **Practical Notes on Diagnosis and Treatment** | 286 |
| **Obstetrics—** | |
| The Relation of Pregnancy to Fibromyomata of the Uterus | 287 |
| **The Royal Army Medical Corps Section—** | |
| The General Hospitals of the Territorial Army | 288 |
| **Anæsthetics—** | |
| Anæsthetisation for Operations on the Central Nervous System | 289 |
| **The General Practitioner's Column—** | |
| How to See a Post-Nasal Space | 290 |
| **Therapeutics—** | |
| Prescriptions and Dispensing | 291 |
| **Resident Medical Officers' Department—** | |
| A Case of Tubercular Tumour of the Cerebellum | 292 |
| **Hospital Administration—** | |
| Graduate Study on the Continent—Germany | 293 |
| Social and Poor-law Problems | 294 |
| The Law of Hospitals | 294 |
| News and Coming Events | 295 |
| **Nursing Administration—** | |
| The Bedding and Linen Department—VII. | 297 |
| **The Graduates' Medical Schools** | 298 |

